# Phenotypic Evaluation of Rare Cystic Fibrosis Transmembrane Conductance Regulator Mutation Combinations in People with Cystic Fibrosis in Queensland, Australia

**DOI:** 10.3390/jcm13206210

**Published:** 2024-10-18

**Authors:** Ieuan Edward Shepherd Evans, Michelle Wood, Vanessa Moore, David William Reid

**Affiliations:** 1 Department of Thoracic Medicine, Adult Cystic Fibrosis Centre, The Prince Charles Hospital, Brisbane, QLD 4032, Australia; 2 Faculty of Medicine, University of Queensland, Brisbane, QLD 4006, Australia; 3 Lung Inflammation & Infection, QIMR Berghofer Medical Research Institute, Brisbane, QLD 4006, Australia

**Keywords:** cystic fibrosis, CFTR genotypes, CF phenotypes, rare mutations

## Abstract

**Background:** Cystic fibrosis (CF) is a multisystem disorder caused by mutations in the cystic fibrosis transmembrane conductance regulator (CFTR) gene. We describe the distribution of CFTR mutation profiles in sub-tropical Queensland, Australia, and characterise the phenotypes associated with ‘rare’ CFTR mutation combinations. **Methods:** We conducted a retrospective observational study to analyse the CFTR mutation profiles of 322 people with CF (pwCF) under the care of a large adult CF centre in Queensland, Australia. Molecular pathology results were available for all identifiable CFTR mutations. The CFTR2 database was utilised to characterise the less common CFTR mutations to define mutation classes and explore associated phenotypic sequelae. **Results:** In total, eighty-seven different genotypes were identified within our CF cohort, with the most abundant mutation being the F508del mutation, 298/322 (92.5%). Thirty-six pwCF with *CFTR* mutations are considered to have ‘rare’ CFTR mutations, and eleven with previously undefined phenotypes. For these eleven pwCF, late diagnosis in adulthood was confirmed in 5/11 pwCF (45.5%) with CFTR modulator therapy only initiated in 5/11 (45.5%). **Conclusions:** The profile of more common *CFTR* genotypes within our cohort of adult pwCF living in Queensland, Australia, generally reflects the global predominance of F508del, G542X, G551D, N1303K, and R117H. The phenotypic heterogeneity of disease seen within the eleven pwCF in our cohort with previously undefined CFTR genotypes highlights that rare mutations can also be associated with severe disease and continue to be at risk of delayed diagnosis. Access to CFTR modulator therapies for this group of pwCF remains limited and should remain a research priority.

## 1. Introduction

Cystic fibrosis is a multisystem disorder caused by mutations in the cystic fibrosis transmembrane conductance regulator (CFTR) gene. The disease is characterised by suppurative lung disease accompanied by pancreatic insufficiency and a host of other extra-pulmonary clinical manifestations. To date, there are over 2000 different CFTR mutations identified with 719 confirmed to be associated with the development of CF [1,2]. Comprehensive annotation of 804 of these CFTR mutations (April 2023) is available from the CFTR2 online global database hosted by Johns Hopkins University and supported by the US Cystic Fibrosis Foundation [2]. The introduction of newborn screening for CF has increased the rate of early detection and, coupled with aggressive management strategies for pulmonary infections and optimised nutritional status, has resulted in improved clinical outcomes. Early interventions to reduce the acquisition of complex respiratory pathogens, such as *Pseudomonas aeruginosa*, and to slow down or prevent the development of disease hallmarks, namely bronchiectasis, have become increasingly available. The advent of CFTR modulator therapies dependent on the underlying CFTR mutation profile, together with advances in molecular detection of CFTR gene mutations, has increased the importance of molecular characterisation and the need for a better understanding of the functional outcome of each CFTR mutation.

Despite improvements in the ability to identify less common/rare CFTR gene mutations, challenges persist around the diagnosis and subsequent management of individuals who present with non-classical CFTR mutation combinations. These individuals may present later in life, sometimes in adulthood because of delayed diagnosis and are often not eligible for current CFTR modulator therapy owing to their rarer mutation combinations [3]. The interplay between genotype and phenotype often results in a varied clinical presentation, even in people with CF (pwCF) who have ‘classical’ CFTR mutation combinations. However, with certain rarer CFTR mutation combinations, disease manifestations are often atypical and frequently distinct from ‘classical’ disease caused by more common CFTR mutations, such as F508del [4]. The contribution of geography and historical migration to the prevalence of less common/rare CFTR genotypes has not been widely studied. However, historical migration patterns are likely to increase the chances of uncommon combinations of CFTR mutations emerging, particularly with migration from the ‘Old World’ to countries such as Australia. The migration patterns to Australia, therefore, provide the potential for an increase in the prevalence of rarer CFTR genotypes to occur, with the occurrence of clinical phenotypes not previously described.

In this study, we describe the distribution of CFTR mutation profiles in sub-tropical Queensland, Australia, and characterise the phenotypes associated with ‘rare’ CFTR mutation combinations. Specific focus is given to highlighting the diversity of CFTR mutations and the phenotypic profile of nine unique CFTR mutation combinations not currently described in global databases.

## 2. Methods

We conducted a retrospective observational study to analyse the CFTR mutation profiles of pwCF managed by the Adult Cystic Fibrosis Centre (ACFC) at The Prince Charles Hospital (TPCH), the largest adult CF centre in Queensland, providing care to 322 pwCF. Diagnoses of CF were made according to the consensus guidelines from the Cystic Fibrosis Foundation [5]. Demographic data were collected for all pwCF in the analysis, including CFTR genotype; baseline forced expired volume in one second (FEV1); sweat chloride (mmol/L); diagnosis of pancreatic insufficiency; diagnosis of CF-related diabetes; diagnosis of CF-related liver disease; HbA1c, bone mineral density (BMD) results; microbiological status; and CFTR modulator prescription. FEV1 was collected as part of spirometry measurements carried out as part of routine clinical care performed on standardised equipment maintained at the ACFC, TCPH. Sweat chloride levels (where available) were checked using the standard method of pilocarpine applied to the skin along with the application of an electrical current to stimulate sweating. Molecular pathology results were available for all identifiable CFTR mutations for the majority of pwCF actively managed by the ACFC. The primary assay utilised was the bi-directional Sanger sequencing of PCR-amplified genomic DNA designed to screen the entire CFTR coding region (exons 1 to 27 plus intron 22 regions 11990–12444). PwCF with alleles not currently identified despite CFTR sequencing were excluded from the analysis. The diagnosis of CF had been made previously in most individuals, usually during Paediatric care or at the time of initial referral to the adult service. A very small number of pwCF was identified in adulthood by clinicians in the ACFC when conducting general respiratory clinics. The CFTR2 database was utilised to further explore the less common CFTR mutations to define mutation classes and the associated phenotypic sequelae. Ethical approval for the study was obtained from the Metro North Health Human Research Ethics Committee (HREC/2022/MNHB/85439).

## 3. Results

### 3.1. CFTR Mutation Profiles

In total, eighty-seven different genotypes were identified within our CF cohort. The most abundant mutation seen in our cohort is the F508del mutation, which is expected given its predominance globally. Within our CF cohort, 298/322 (92.5%) of pwCF possessed an F508del CFTR mutation; 141/322 (43.8%) were homozygous and 157/322 (48.8%) were heterozygous for this mutation. 

Other CFTR mutations with >1% predominance within the cohort included: G551D (33/322, 10.2%—with two pwCF homozygous for the G551D CFTR mutation); R117H, either unknown poly T tract, 5T, 7T or 9T, (20/322, 6.2%); G542X (11/322, 3.4%); N1303K (8/322, 2.5%), A455E (6/322, 1.9%); 621+1G->T (6/322, 1.9%), I507del (5/322, 1.6%); and Q1291H, 1717-1G->A, 1154insTC, 2184delA, D1152H, R553X (4/322, 1.2%). Further descriptions of these ten most frequently encountered CFTR mutations are provided in Table 1.

**Figure 1:**
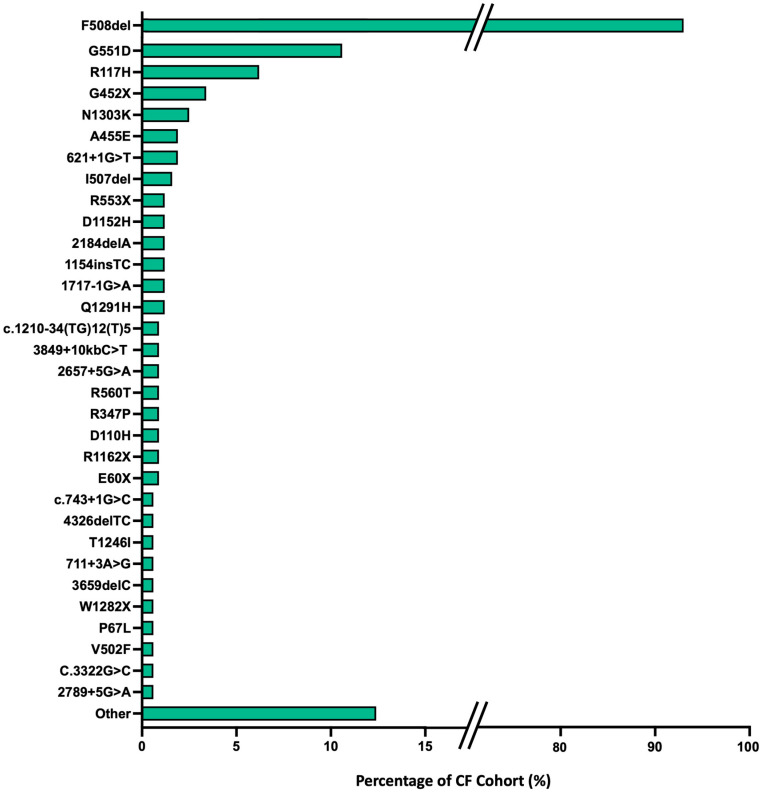
Full list of CFTR mutation variants encountered within the TPCH CF cohort with the predominant CFTR mutations seen in ≥2 pwCF highlighted. The remaining mutations are collated as ‘other’. F580del mutations predominate (43.8% homozygous, 48.8% heterozygous) with G551D the second most prevalent mutation (0.6% homozygous, 9.6% heterozygous).

The full spectrum of CFTR mutation profiles from the TPCH CF cohort is detailed in Figure 1.

### 3.2. Geographic Origin of CFTR Mutations

**Figure 2:**
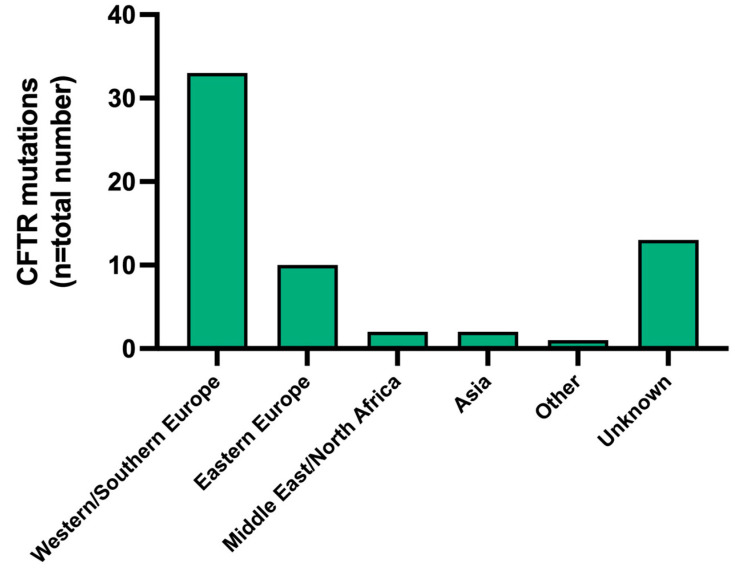
Geographic origins of *CFTR* mutations in pwCF under the care of the ACFC at TPCH [6,7,8,9,10,11,12,13,14,15,16,17,18,19,20,21,22,23,24,25,26].

Migration patterns to Australia over the past 235 years, especially since the 1945 post-World War 2 immigration drive, have impacted on the diversity of CF genotypes encountered in clinical practice. Establishing the geographic origins of CFTR mutations, where possible, was attempted, as illustrated in Figure 2. The highest proportion of CFTR mutation frequencies within our cohort were derived, as expected, from European populations. 

Taking into consideration the geographic predominance of CFTR mutations originating in Europe, particularly Western and Southern Europe, a comparison was made between the allelic frequencies of the most common CFTR variants found in the ACFC cohort to that of the European Cystic Fibrosis Society (ECFS) annual report, 2022. In total, seven of the most prevalent mutations found in pwCF under the care of the ACFC also predominated in the ECFS database, including F508del (Denmark 82.5%), G551D (Ireland 8.3%), R117H (Ireland 3.1%), G542X (Armenia 8.0%), N1303K (Iceland 40.0%), 621+1G>T (Greece 6.9%), and 1717-1G>A (Switzerland 2.7%) [27].

### 3.3. Rare CFTR Mutation Combinations

As discussed, the geographical heterogeneity in CFTR mutation origins has the potential to contribute to the increased chance of rare CFTR mutation combinations arising. Affected individuals may have previously undefined and unusual CF disease phenotypes. For the purposes of this analysis, we have focused on those mutation profiles with <50 pwCF documented globally within the CFTR2 database. Within our cohort, there were 36 pwCF with CFTR mutations considered to be rare and of these, eleven pwCF had previously undefined phenotypes, Table 2.

The eleven pwCF identified with previously uncharacterised CFTR mutation combinations included two sets of pwCF with the same CFTR mutation combination based on the familial connection (F508del/V1180L (c.3322delG) and G551D/D110H). The unique phenotypes of these CFTR genotypes are further defined in Table 3A.

Late diagnoses of CF in adulthood were confirmed from medical records in 5/11 pwCF (45.5%). The remaining six pwCF were diagnosed in childhood. However, restricted access to paediatric notes prohibited confirmation of exact timings of diagnosis. The pwCF who had their diagnoses made in adulthood had the following mutation profiles: P67L/3272-26A->G; W1282X/I1269N; G542X/T1246I; E60X/Q1291H; and V520F/1461ins4.

Pre-CFTR modulator sweat chloride levels were available for 6/11 (54.5%) of the pwCF with previously undefined phenotypes with all of them recording values of <60 mmol/L. Phenotypic variability is highlighted by FEV_1_ (% predicted), particularly in those pwCF with familial connection (F508del/V1108L and G551D/D110H). Nine (81.8%) of pwCF had a diagnosis of pancreatic insufficiency with 3/11 (27.2%) diagnosed with CFRD and one pwCF exhibiting an indeterminate OGTT result. Two pwCF had diagnoses of osteoporosis and 4/11 (36.3%) showed features of osteopenia on BMD testing. Microbiological colonisation of sputum showed a predominance of *P. aeruginosa* and *S. aureus*. CFTR modulator therapy was initiated with 5/11 (45.5%) of pwCF with only two pwCF identified as having an F508del genotype valid for prescription of elexacaftor/tezacaftor/ivacaftor (ETI, Vertex Pharmaceuticals) under the current Pharmaceutical Benefits Scheme (PBS) in Australia.

Within our CF cohort, there were also three pwCF with mutation profiles classed as ‘non-disease causing’ by the CFTR2 database. These include pwCF with the following CFTR mutation combinations: F508del/I148T; F508del/M470V; and F508del/I1027T. The phenotypes for these pwCF are described in Table 3B.

## 4. Discussion

The profile of more common CFTR genotypes within our cohort of adult pwCF living in Queensland, Australia, generally reflects the global predominance of F508del, G542X, G551D, N1303K, and R117H [28]. These predominant mutations have their geographic origins in Western and Southern Europe and are consistent with the countries of origin of migration patterns to Australia since 1788 when the first European colonisation of Australia occurred. The initial waves of European migrants were predominantly formed of people of Dutch, Irish, Scottish and English heritage. Subsequent migration remained largely limited to countries from Europe owing to the Naturalisation Act of 1903 right up until its abolition in 1966. In more recent times, Australia has seen increasing numbers of people migrating from wider global origins, such as Eastern Europe, Southeast Asia, and New Zealand, with smaller contributions from North America and Africa. This, combined with an Aboriginal population that inhabited Australia prior to early European settlement, has potentially added to the diversity of the CFTR mutation pool in our current cohort. 

In our cohort, thirty-six pwCF were identified as having rare mutations (as defined by <50 pwCF recognised globally in the CFTR2 database). Of these pwCF, eleven had CFTR mutation profiles with associated phenotypes not previously described in the literature or defined by the CFTR2 database. There is a significant degree of heterogeneity in the phenotypes exhibited by these mutation combinations, with the role of environmental or other genetic modifiers impact evidenced by the significant phenotypic variability noted in two sibling pairs of pwCF with identical genotypes. 

In addition to the wide variability in disease phenotype in those pwCF with rare mutations, there was also significant variability in sweat chloride results, although all were below <60 mmol/L, the level above which CF would traditionally be considered. Whilst already well established, this serves as a timely reminder that sweat chloride alone should not be relied upon to diagnose CF, but rather should be used in correlation with genetic screening, clinical assessment and multidisciplinary team assessment, especially in the setting of rare CFTR genotypes. Furthermore, one of the pwCF with a genotype not considered to be disease causing, F508del/I148T, recorded a sweat chloride level of 110 mmol/L highlighting the variability in reliability, and the disconnect that may occur, between sweat chloride results and both CF genotype and phenotype.

Rare CF genotypes are often thought to be reflected by milder phenotypes, a feature felt to occasionally contribute to diagnosis in adulthood. However, the overall phenotypic profile for the eleven pwCF in our cohort with previously undefined CFTR mutation combinations overall exhibited reasonably severe disease with FEV_1_ (% predicted) values ranging between 35.4 and 104.4%. Additionally, extra-pulmonary manifestations of CF were relatively high with pancreatic insufficiency in nearly all individuals (81.8%), CFRD present in approximately a quarter (27.2%) and osteoporosis/osteopenia apparent in just over half (54.5%). Interestingly, liver disease was present in only one pwCF and this was mild with ultrasound changes consistent with hepatic steatosis. Lung microbiology was also consistent with classical CF-related bacterial pathogens, such as *P. aeruginosa* and *S. aureus*, present in most individuals, although *Aspergillus fumigatus* was only isolated in the two related pwCF with the F508del/V1108L genotype.

The other challenge that will be a universal one in CF centres is how to manage pwCF with rarer CFTR genotypes who are currently not eligible for CFTR modulator therapy based on their mutation profile [29]. Clinical trials did not recruit pwCF with rare mutations unless they were compound heterozygotes for a F508del mutation and, even then, some individuals would not have been eligible because their allelic mutation may not have been characterised as a minimal function, gating or residual function mutation. Anecdotally, we have observed clinical benefits in pwCF commenced on ETI who have an F508del paired with a rare or ‘non-disease causing’ CFTR mutation but whether pwCF with a combination of rare CFTR mutations would respond is not currently known. Certainly, within our eleven pwCF with rare CFTR mutations, only two had an F508del genotype combination eligible for ETI under the current PBS criteria in Australia, with just over half exhibiting genotypes not suitable for any currently available CFTR modulator therapy (54.5%).

Finally, it is worth considering the three pwCF within our cohort with mutation combinations determined to be ‘non-disease causing’ by the CFTR2 database. The combination of F508del/I148T has been determined to potentially exhibit a disease phenotype with mild symptoms in selected organs and/or be associated with the diagnosis of a ‘CFTR-related disorder’. However, it is not expected to result in symptoms and features that fulfil the diagnostic criteria for CF. Similarly, the F508del/M470V genotype is again not considered disease causing. The M470V variant is very common within the general population, with approximately 50% of people possessing this variant. Individuals who are homozygous for the M470V CFTR variant do not appear to have a CF phenotype. The I1027T is a complex allele and would usually be determined not to cause disease, even when combined with a disease-causing mutation. However, all three of these pwCF exhibited clinical phenotypes in keeping with CF, including one abnormal sweat chloride test. These observations reinforce the importance of considering phenotypic characterisation in the diagnosis of CF as well as all the other aspects, such as genotype and sweat chloride results, rather than excluding the diagnosis based on any one specific aspect in isolation. It is also worth noting alternative methods of diagnosis that could be implemented for such pwCF with either inconclusive sweat chloride levels or CFTR genetic testing results. Diagnostic tests, including nasal potential difference, which are used to measure the voltage across the nasal epithelium as a surrogate for CFTR function, and intestinal current measurements utilising rectal biopsies to carry out ex vivo testing in a Ussing chamber for electrical responses to various secretagogues, may be considered [30,31]. Neither of these testing modalities was carried out for any pwCF in our cohort.

## 5. Conclusions

The phenotypic heterogeneity of disease seen within the eleven pwCF in our cohort with previously undefined CFTR genotypes highlights that rare mutations can also be associated with severe disease, irrespective of whether they are diagnosed late. These mutation combinations remain a prominent focus of ongoing research, specifically from a CFTR modulator perspective, given the relative paucity of currently available treatment options. This is true for the eleven pwCF in our cohort with only two pwCF being suitable for the CFTR modulator therapy, ETI. The importance of thorough evaluation of clinical phenotype when making a diagnosis is reflected well in our cohort, especially taking into consideration the lack of diagnostic sweat chloride levels in all but one of the pwCF analysed. Whilst the diagnosis of CF can often be challenging, improvements in diagnostics, including CFTR gene sequencing methodologies [32], should aid with earlier detection and thereby earlier implementation of directed therapies, albeit with the caveat that ongoing work around new therapeutic options for this group of pwCF should remain a research priority.

## Figures and Tables

**Table 1:** CF cohort–ten most frequently encountered CFTR mutations.

Mutation	Nucleotide Change	Legacy Name	refSNP	Variant Type	Cohort Proportion
p.Phe508del	c.1521_1523del	F508del	rs113993960	Deletion (3 nucleotides)	Homozygote—141 (43.8%)
					Heterozygote—157 (48.8%)
p.Gly551Asp	c.1652G>A	G551D	rs75527207	Missense—risk allele A	Homozygote—2 (0.6%)
					Heterozygote—31 (9.6%)
p.Arg177His	c.350G>A	R117H	rs78655421	Missense—risk allele A	Heterozygote—20 (6.2%)
p.Gly543X	c.1624G>T	G542X	rs113993959	Nonsense—risk allele T	Heterozygote—11 (3.4%)
p.Asn1303Lys	c.3909C>G	N1303K	rs80034486	Missense—risk allele G	Heterozygote—8 (2.5%)
p.Ala455Glu	c.1364C>A	A455E	rs74551128	Missense—risk allele A	Heterozygote—6 (1.9%)
p.?	c.489+1G>T	621+1G>T	rs78756941	Variant to splice donor/acceptor site	Heterozygote—6 (1.9%)
p.Ile507del	c.1519_1521delATC	I507del	rs121908745	Frame insertion/deletion	Heterozygote—5 (1.6%)
p.Gln1281His	c.3873G>C	Q1291H	rs121909015	Single nucleotide variant	Heterozygote—4 (1.2%)
p.?	c.1585-1G>A	1717-1G>A	rs76713772	Variant to splice donor/acceptor site	Heterozygote—4 (1.2%)

? = current unknown mutations.

**Table 2:** TPCH CF cohort—rare CFTR mutations with unrecorded phenotypic characterisation. Functional CFTR testing data have been extracted from the CFTR2 database [2].

CFTR Mutation	Functional Testing CFTR Mutation 1	Functional Testing CFTR Mutation 2	Global Database Records (CFTR2)
P67L/3272-26A->G	Missense	Missense	Unknown combination
W1282X/I1269N	Nonsense	Missense	Unknown combination
F508del/V1108L (c.3322delG) *	Frame insertion/deletion	Unknown	Unknown combination
G542X/T1246I	Nonsense	Missense	Unknown combination
1898+1G->A (1766+1G>A)/2622 + 1G->T (2490+1G>T)	Unknown	Variant to splice donor/acceptor site	Unknown combination
E60X/Q1291H	Nonsense	Nonsense	Unknown combination
G551D/D110H *	Missense	Missense	Unknown combination
1249-1G>A (c.1117-5A>G)/c.1210-34(TG)12(T)5	Variant to splice donor/acceptor site	Unknown	Unknown combination
V520F/1461ins4	Missense	Insertion/deletion variant	Unknown combination

* = these genotypes were present in two sets of pwCF.

**Table 3:** (**A**) Disease phenotypes associated with CFTR mutation combinations not currently described in the literature. (**B**) Disease phenotypes for pwCF with CFTR mutation combinations reported to be ‘non-disease causing’ in the CFTR2 database.

**(A) Rare CFTR Mutation Combinations—Phenotypes**										
**CFTR Genotype**	**Baseline FEV1**	**BMI**	**Sweat** **Chloride** **(mmol/L)**	**Pancreatic** **Insufficiency**	**CFRD**	**CFLD**	**HbA1c**	**Bone Health (BMD)**	**Microbiology**	**CFTR Modulator**
P67L/3272-26A->G	1.38 (52.1%)	29.4	51	Yes	No	No	5.5%	Osteoporosis	PsA, *S. aureus*	Tezacaftor/ivacaftor
W1282X/I1269N	1.65 (52.2%)	35.8	N/A	Yes	No	No	5.30%	Osteoporosis	PsA (intermittent)	Nil
F508del/V1108L (c.3322delG)	3.75 (103.4%)	21.8	N/A	Yes	No *	No	5.70%	Normal	PsA, *S. aureus*, *Asp. fumigatus*	Elexacaftor/tezacaftor /ivacaftor
F508del/V1108L (c.3322delG)	2.37 (68.5%)	19.4	N/A	Yes	Yes	Yes	10.60%	Osteopenia	PsA, *S. aureus*, *Asp. fumigatus*	Elexacaftor/tezacaftor /ivacaftor
G542X/T1246I	1.25 (47.6%)	24.3	53	No	No	No	5.80%	Normal	PsA	Nil
1898+1G->A (1766+1G>A)/2622+1G->T (2490+1G>T)	3.04 (95.0%)	26.1	N/A	Yes	Yes	No	5.80%	Normal	PsA, *S. aureus*	Nil
E60X/Q1291H	0.95 (35.4%)	28.6	49	Yes	Yes	No	8.50%	Osteopenia	PsA, *S. aureus*	Nil
G551D/D110H	2.61 (63.2%)	24.7	28	Yes	No	No	5.30%	Osteopenia	Nil regular	Ivacaftor
G551D/D110H	4.03 (104.4%)	22.2	23	Yes	No	No	5.00%	Osteopenia	Nil regular	Ivacaftor
1249-1G>A (c.1117-5A>G)/c.1210-34(TG)12(T)5	2.55 (54.5%)	30.7	57	No	No	No	N/A	No BMD	Nil regular	Nil
V520F/1461ins4	2.90 (67.6%)	23.6	N/A	Yes	No	No	6.10%	Normal	*S. aureus*	Nil
**(B) Non-disease Causing CFTR Mutation Combinations—Phenotypes**										
**CFTR Genotype**	**Baseline FEV1**	**BMI**	**Sweat** **Chloride** **(mmol/L)**	**Pancreatic** **Insufficiency**	**CFRD**	**CFLD**	**HbA1c**	**Bone Health** **(BMD)**	**Microbiology**	**CFTR Modulator**
F508del/I148T	2.79 (67.8%)	20.9	110	Yes	No *	No	5.30%	Osteopenia	*S. aureus*	Elexacaftor/tezacaftor /ivacaftor
F508del/M470V	0.94 (24.1%)	34.3	43	Yes	Yes	Yes	9.00%	Osteopenia	PsA	Elexacaftor/tezacaftor /ivacaftor
F508del/I1027T	1.49 (39.2%)	26.4	N/A	Yes	No	Yes	5.00%	Normal	PsA	Elexacaftor/tezacaftor /ivacaftor

OGTT—oral glucose tolerance test (* impaired glucose tolerance); CFRD—CF-related diabetes; CFLD—CF-related liver disease; BMD—bone mineral density scan; PsA—*Pseudomonas aeruginosa*; *S. aureus*—*Staphylococcus aureus*; *Asp. fumigatu*—*Aspergillus fumigatus*.

## Data Availability

No publicly available data available.
